# 3D Collagen-Nanocellulose Matrices Model the Tumour Microenvironment of Pancreatic Cancer

**DOI:** 10.3389/fdgth.2021.704584

**Published:** 2021-07-26

**Authors:** Rodrigo Curvello, Verena Kast, Mohammed H. Abuwarwar, Anne L. Fletcher, Gil Garnier, Daniela Loessner

**Affiliations:** ^1^Department of Chemical Engineering, Faculty of Engineering, Monash University, Clayton, VIC, Australia; ^2^Max Bergmann Center of Biomaterials Dresden, Leibniz Institute of Polymer Research Dresden E.V., Dresden, Germany; ^3^Department of Biochemistry and Molecular Biology, Biomedicine Discovery Institute, Monash University, Clayton, VIC, Australia; ^4^Department of Chemical Engineering, Bioresource Processing Research Institute of Australia (BioPRIA), Monash University, Clayton, VIC, Australia; ^5^Department of Materials Science and Engineering, Faculty of Engineering, Monash University, Clayton, VIC, Australia; ^6^Department of Anatomy and Developmental Biology, Biomedicine Discovery Institute, Faculty of Medicine, Nursing and Health Science, Monash University, Clayton, VIC, Australia

**Keywords:** pancreatic cancer, nanocellulose, collagen, hydrogels, extracellular matrix, stiffness

## Abstract

Three-dimensional (3D) cancer models are invaluable tools designed to study tumour biology and new treatments. Pancreatic ductal adenocarcinoma (PDAC), one of the deadliest types of cancer, has been progressively explored with bioengineered 3D approaches by deconstructing elements of its tumour microenvironment. Here, we investigated the suitability of collagen-nanocellulose hydrogels to mimic the extracellular matrix of PDAC and to promote the formation of tumour spheroids and multicellular 3D cultures with stromal cells. Blending of type I collagen fibrils and cellulose nanofibres formed a matrix of controllable stiffness, which resembled the lower profile of pancreatic tumour tissues. Collagen-nanocellulose hydrogels supported the growth of tumour spheroids and multicellular 3D cultures, with increased metabolic activity and matrix stiffness. To validate our 3D cancer model, we tested the individual and combined effects of the anti-cancer compound triptolide and the chemotherapeutics gemcitabine and paclitaxel, resulting in differential cell responses. Our blended 3D matrices with tuneable mechanical properties consistently maintain the growth of PDAC cells and its cellular microenvironment and allow the screening of anti-cancer treatments.

## Introduction

The tumour microenvironment (TME) is a complex and dynamic niche that integrates the extracellular matrix (ECM), multiple cell populations and an array of secreted factors and signalling molecules (1). Despite our improved understanding about the role of the TME in cancer progression, pancreatic ductal adenocarcinoma (PDAC) has one of the highest cancer-related mortality rates, with a 5-year survival rate of 10% (2–4). This is caused by the late diagnosis of the disease, multi-drug resistance, and lack of targeted therapies, which diminishes the efficacy of existing treatments and hinders the development of new treatments. In this context, PDAC models have been applied to screen anti-cancer or anti-metastatic therapeutics and to study and better understand tumour biology. There is a vast literature not only for pancreatic cancer models, but also for breast and prostate tumours based on animal, two-dimensional (2D), and three-dimensional (3D) systems (1). Whilst traditional 2D cell monolayers do not recapitulate the complexity of tumour tissues and animal models are limited in their reproducibility and costly (5), 3D models are controllable and reproducible tools for cancer research (4, 6). Advances in tissue engineering technologies enabled scientists to deconstruct and mimic individual components of the TME, emulating the dynamics arising from the diverse extracellular and cellular elements (7, 8). Several studies have demonstrated the application of 3D approaches to model pancreatic tumours and responses to treatment (9–12). For example, the incorporation of key ECM elements, such as collagens and hyaluronan, indicated the complex matrix composition and the impact of ECM remodelling (13, 14). The cellular and signalling interactions were integrated by culturing pancreatic cancer cells together with key stromal cells, such as cancer-associated fibroblasts (CAFs), endothelial cells, and immune cells (15). The modelling of the pancreatic TME shed also light on the immunological dysregulation and the abnormal metabolic signature, two important hallmarks of cancer (16, 17). However, the continuous advancement of ECM-like biomaterials, such as hydrogels, that closely reproduce the specific features of tumour tissues is critical for biomimetic 3D matrices to model the pancreatic TME.

Hydrogels are aqueous materials with tuneable mechanical and chemical properties and have been used to encapsulate cancer cells as they mimic the physiological features of tumour tissues seen in patients (18). Most hydrogels that are commercially available do not replicate the matrix properties of PDAC, which has a stiff and fibrotic profile varying from about 2 to 6 kPa (19–21). Natural hydrogels made of collagen and laminin-rich decellularized matrices, the most common options for 3D cell cultures, are restricted by their poor mechanical properties that vary between 100 and 400 Pa (22). To address this limitation, synthetic hydrogels made of inert polymeric chains or bioactive macromolecules have emerged as ECM-like biomaterials to engineer the TME (23, 24). Among those is cellulose, a biocompatible carbohydrate polymer abundantly found in plants and bacteria. Cellulose-based hydrogels have attracted attention amongst biologists (25). These hydrogels are formed by short nanocrystals or elongated nanofibres of 1-5 nm in diameter, referred to as nanocellulose, and are functionalized with cell adhesive proteins for enhanced cell-surface interactions. Nanocellulose matrices have been used in a variety of 3D cancer models (Table 1).

**Table 1:** Examples of cellulose hydrogels applied as 3D cancer models.

**Type**	**Functionalization**	**Cancer type**	**Cell line**	**Treatment**	**References**
Cellulose nanocrystals	Grafting of poly-NiPAAm for thermo-responsiveness	Breast	MCF-7	None	Li et al. (26)
	Grafting of hexadecyl-amine for pH-responsiveness	Hepatic, lung	HepG2, A549	Paclitaxel	Ning et al. (27)
Cellulose nanofibres	None	Hepatic	HepG2	None	Auvinen et al. (28)
	None	Breast	MDA-MB-231, T-47D	None	Barnawi et al. (29)
	Blending with UV-responsive hemicellulose	Pancreatic	SW-1990	None	Xu et al. (30)
Bacterial nanocellulose	Grafting of laminin-derived (IKVAV) peptides	Melanoma	SK-MEL-28	None	Reis et al. (31)
	Labelling with pH-sensitive proteins	Colon	HCT116	None	O'Donnell et al. (32)
	Blending with gelatin	Glioblastoma	U251 MG	None	Unal et al. (33)

From a bioengineering perspective, nanocellulose is a low cost and sustainable fibrous scaffold to mimic the architecture of the ECM. Our team used nanocellulose hydrogels as tailorable matrices for the growth of small intestinal organoids (34). We also demonstrated the effect of blending type I collagen with nanocellulose to form hydrogels of adjustable stiffness for 3D tissue models (35). The strong affinity of collagen to nanocellulose, together with the predominance of this ECM protein in PDAC tissues (36), make collagen-nanocellulose hydrogels a promising candidate to engineer the TME. We hypothesised that collagen-nanocellulose matrices are a renewable and controllable biomaterial to develop a 3D pancreatic cancer model.

**Figure 1:**
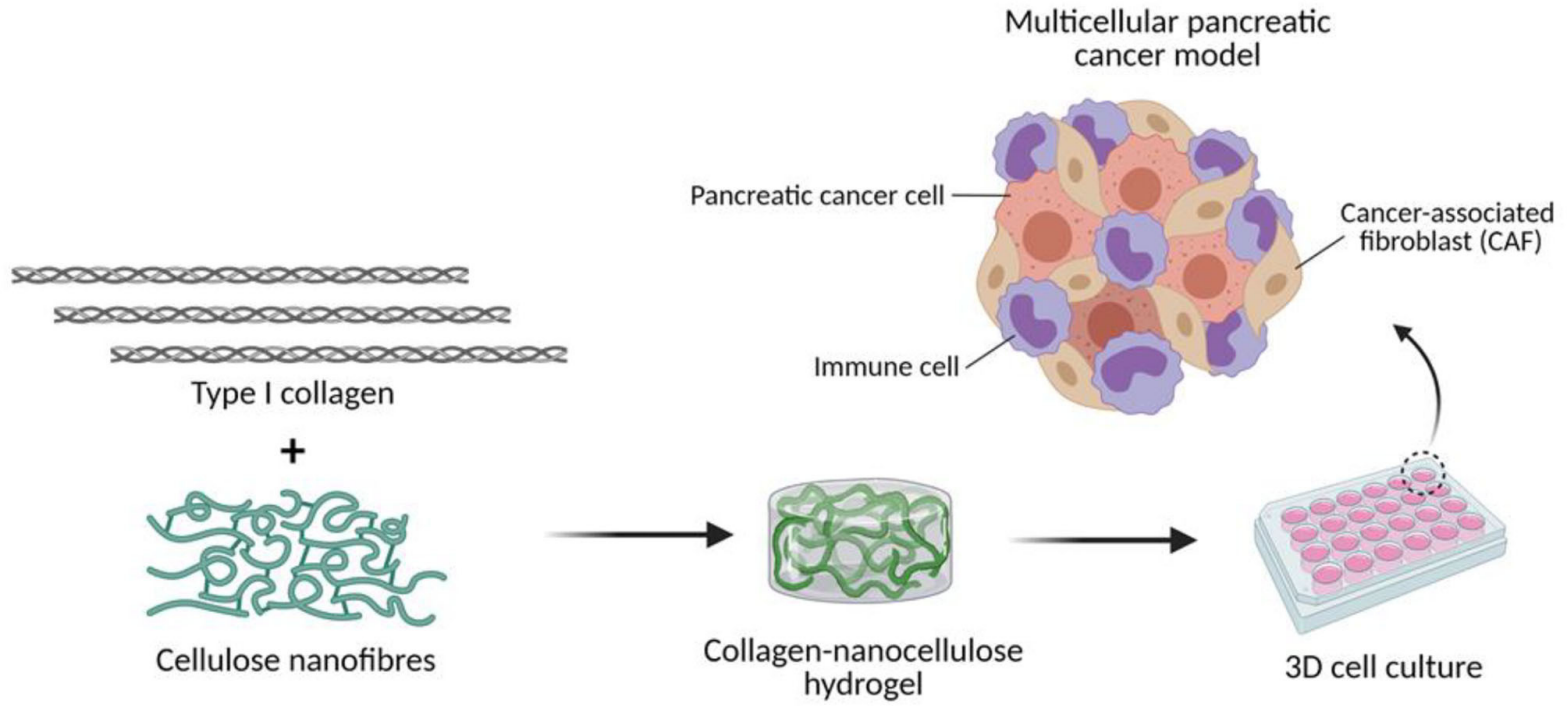
Engineering the TME of pancreatic cancer with collagen-nanocellulose hydrogels. Type I collagen fibrils and cellulose nanofibres are blended to form hydrogels that support the growth of multiple cell populations found in pancreatic cancer tissues.

In this study, we investigated the suitability of collagen-nanocellulose matrices to recapitulate the extracellular and cellular elements of the pancreatic TME (Figure 1). Once reinforced with nanocellulose, collagen hydrogels achieved a stiffness within the lower range of PDAC tissues. PDAC cells grown encapsulated within the hydrogels formed spheroids over 7-14 days of 3D culture and were used to test responses to combined treatment of gemcitabine and paclitaxel with triptolide. While the treatment with triptolide had a strong effect on cell viability, combined treatment with gemcitabine and paclitaxel showed minimal cytotoxic effects. The incorporation of stromal cell populations, such as CAFs and myeloid cells, mimicked the multicellular composition of the pancreatic TME. Multicellular 3D cultures of tumour spheroids with stromal cells displayed up to 2 times higher Young's moduli and responded to triptolide compared to tumour spheroid controls. Our new 3D cancer model may be further explored to decipher the tumour biology of different stromal cell types for the design of improved or new precision therapies.

## Materials and Methods

### Synthesis of Nanocellulose Hydrogels

TEMPO-periodate oxidation of nanocellulose was performed following an established protocol (37). Briefly, 10 g of never dried bleached Eucalyptus Kraft (BEK) pulp (Australian Paper, Maryvale, Australia) was disintegrated and transferred to 1 L of Milli-Q water containing 2,2,6,6-tetramethylpiperidine-1-oxyl (TEMPO) (0.5 mmol/g cellulose) (Sigma-Aldrich 214000), NaIO4 (2.5 mmol/g cellulose) (Sigma-Aldrich 311448), and NaBr (8 mmol/g cellulose) (Sigma-Aldrich 71329). Oxidation was initiated by addition of 12% NaClO (8 mmol/g cellulose) (Sigma-Aldrich 425044), and the pH was maintained at 10.5. Oxidised cellulose fibres were washed with Milli-Q water through vacuum filtration. Carboxylate group content was quantified by conductometric titration (38). Oxidised pulp was disintegrated in Milli-Q water (1.0 wt% solid content) and passed through a high-pressure homogenizer (GEA Niro Soavi Homogeniser Panda) at 700-900 bar.

### Synthesis of Collagen and Collagen-Nanocellulose Hydrogels

Collagen-nanocellulose hydrogels were prepared as previously published (35). Briefly, different volumes of type I collagen solution from bovine skin (0.27 wt%, Sigma-Aldrich C4243) and nanocellulose hydrogels (1 wt%) were combined to achieve the final concentrations desired (Table 2). Hydrogels were sterilised using ultraviolet radiation at a dose of 250 nm for 20 min in a Safemate ECO Class 2 Biological Safety Cabinet before use in 3D cell cultures.

**Table 2:** Collagen-nanocellulose hydrogel formulations.

**Hydrogel name**	**Volume of COL solution (μl)**	**Volume of NC hydrogel (μl)**	**Volume of PBS (μl)**	**COL (final wt%)**	**NC (final wt%)**
COL	750	0	250	0.2	0
COL-NC-01	750	125	125	0.2	0.1
COL-NC-02	750	250	0	0.2	0.2

*COL, collagen; NC, nanocellulose; PBS, phosphate-buffered saline*.

### Mechanical Testing

The mechanical properties of the hydrogels were characterised by rheology. Oscillatory strain sweep measurements (0.01-100% strain, 1 Hz frequency) were performed at 37°C, having a solvent trap to prevent temperature variation. Firstly, a volume of 50 μl of hydrogel was casted on a non-treated 48-well tissue culture plate. Hydrogels were incubated at 37°C, 5% CO_2_ for 40 min to achieve gellification and the formation of discs. Then, hydrogel discs were covered with 500 μl Dulbecco's Modified Eagle Medium (DMEM) (Gibco 11965092) supplemented with 10% foetal bovine serum (FBS, Gibco 10099-141) and 1% penicillin/streptomycin (P/S) and incubated at 37°C, 5% CO_2_ for 3 h. After that, samples were transferred to the rheometer surface (Anton Paar MCR302) for measurement using a parallel plate (PP15, ø = 15 mm, gap = 0.1 mm). The same method was applied to cell-containing hydrogels, which were tested for their mechanical properties after 14 days of 3D culture. The storage modulus in the linear viscoelastic region was adopted as the shear modulus, and the Young's modulus (E) calculated as:


E=2G (1+ υ)

where G is the shear modulus, and υ is the Poisson's ratio (considered as 0.5 for incompressible materials).

### Imaging of the Hydrogel Structure

A volume of 50 μl of collagen and collagen-nanocellulose hydrogels was dispensed on a 25 mm round coverslip and snap frozen at −180°C for 120 s. Then, samples were lyophilized for 3 h at −55°C under vacuum pressure at 0.03 mBar (0.003 kPa) using an Alpha 1-2 LDplus 2.5 L freeze-dryer (Martin Christ, Germany). Samples were sputter coated with gold (Baltec SCD 050, USA) for 300 s and 20 mV, and the morphological ultrastructure visualised by scanning electron microscopy (SEM) using a FEI SEM (Quanta 250) at an emission current of 30 mA and 10 kV voltage (Ramaciotti Centre for Cryo-Electron Microscopy, Monash University, Australia).

### 3D Cell Culture

Human PDAC cell lines MIA PaCa-2 and PANC-1, and acute monocytic leukaemia THP-1 cells were purchased from the European Collection of Authenticated Cell Cultures (ECACC). Human immortalised pancreatic cancer-associated fibroblasts (CAFs) were established by the group of Dr Anne L. Fletcher (39). PDAC cells and CAFs were maintained as adherent monolayers, while THP-1 cells were grown in suspension using DMEM supplemented with 10% FBS and 1% P/S. All cells were routinely tested for mycoplasma. For 3D culture, cells at a 70-80% confluence were used and resuspended in the hydrogels at the desired cell densities (Table 3). Cells were mixed with a volume of 50 μl of hydrogel solution, placed in non-treated 48-well plates to promote hydrogel formation, and incubated at 37°C, 5% CO_2_ for 40 min. Then, a volume of 500 μl DMEM medium supplemented with 10% FBS and 1% P/S was added to each well and renewed every other day. The same cell densities as shown in Table 3 were applied for 2D monocultures of cancer cells. 3D cell cultures were conducted for 14 days and observed by brightfield microscopy.

**Table 3:** Cell seeding density used for 3D cell cultures.

**Type of culture**	**PDAC cells**	**Cell density (cells/ml)**	**Stromal cells**	**Cell density (cells/ml)**
Mono-culture	MIA PaCa-2	2.5 × 10^5^	CAFs and THP-1	1.0 × 10^5^ (each)
	PANC-1	2.5 × 10^5^		
Triple-culture	MIA PaCa-2	0.5 × 10^5^	CAFs and THP-1	1.0 × 10^5^ (each)
	PANC-1	0.5 × 10^5^		

*PDAC, pancreatic ductal adenocarcinoma; CAFs, cancer-associated fibroblasts*.

### Immunofluorescent Staining and Confocal Laser Scanning Microscopy

Cell-seeded hydrogel samples were fixed in 4% paraformaldehyde solution containing 0.2% triton-X 100 (Sigma-Aldrich, T9284) and washed with phosphate-buffered saline (PBS) and 0.1 M glycine solution. Samples were incubated with rhodamine-conjugated phalloidin (200 U/mL, Invitrogen R415) diluted 1:250 in 1% bovine serum albumin in PBS at room temperature for 60 min, protected from light. Samples were washed twice with PBS and counterstained with DAPI (5 mg/ml, Invitrogen D1306) diluted 1:2,000 in PBS for 30 min at room temperature, protected from light. Immunofluorescent signals were detected using an Olympus FV3000 confocal laser scanning microscope.

### Cell Metabolic Activity and Treatment

Metabolic activity was measured on days 1, 7, and 14, and after treatment. One part of prestoblue reagent (Invitrogen A13261) was diluted in nine parts of phenol red-free DMEM (Gibco 21063029). A volume of 200 μl prestoblue solution was added to each well containing cell-seeded hydrogel samples and the cell-free hydrogel controls. Samples were incubated at 37°C, 5% CO_2_ for 45 min, protected from light. A volume of 90 μl prestoblue solution was transferred from each well to a black with clear bottom 96-well plate (Invitrogen 265301) in duplicates. The absorbance was measured using a SpectraMax M2e fluorescent microplate reader (560 nm excitation, 590 nm emission). To detect responses to treatment, cell-seeded hydrogels were treated with 25 nM triptolide (Sigma-Aldrich T3652) on day 7, followed by the treatment with 100 nM gemcitabine (Sigma-Aldrich G6423) and 100 nM paclitaxel (Sigma-Aldrich T7402) on both days 10 and 12. On day 14, the metabolic activity was measured and normalised to the untreated controls per group.

### Statistical Analysis and Diagram Drawing

Differences were assessed by two-way ANOVA test followed by the Tukey's multiple comparison test in GraphPad Prism 8. Results for all analyses with a *p*-value lower than 0.05 were considered to indicate statistically significant differences (^*^ = *p* ≤ 0.05; ^**^ = *p* ≤ 0.01; ^***^ = *p* ≤ 0.001). The diagram shown in Figure 1 was created using Biorender.com.

## Results

### Mechanical and Cellular Prerequisites for 3D Cultures Using Collagen-Nanocellulose Hydrogels

**Figure 2:**
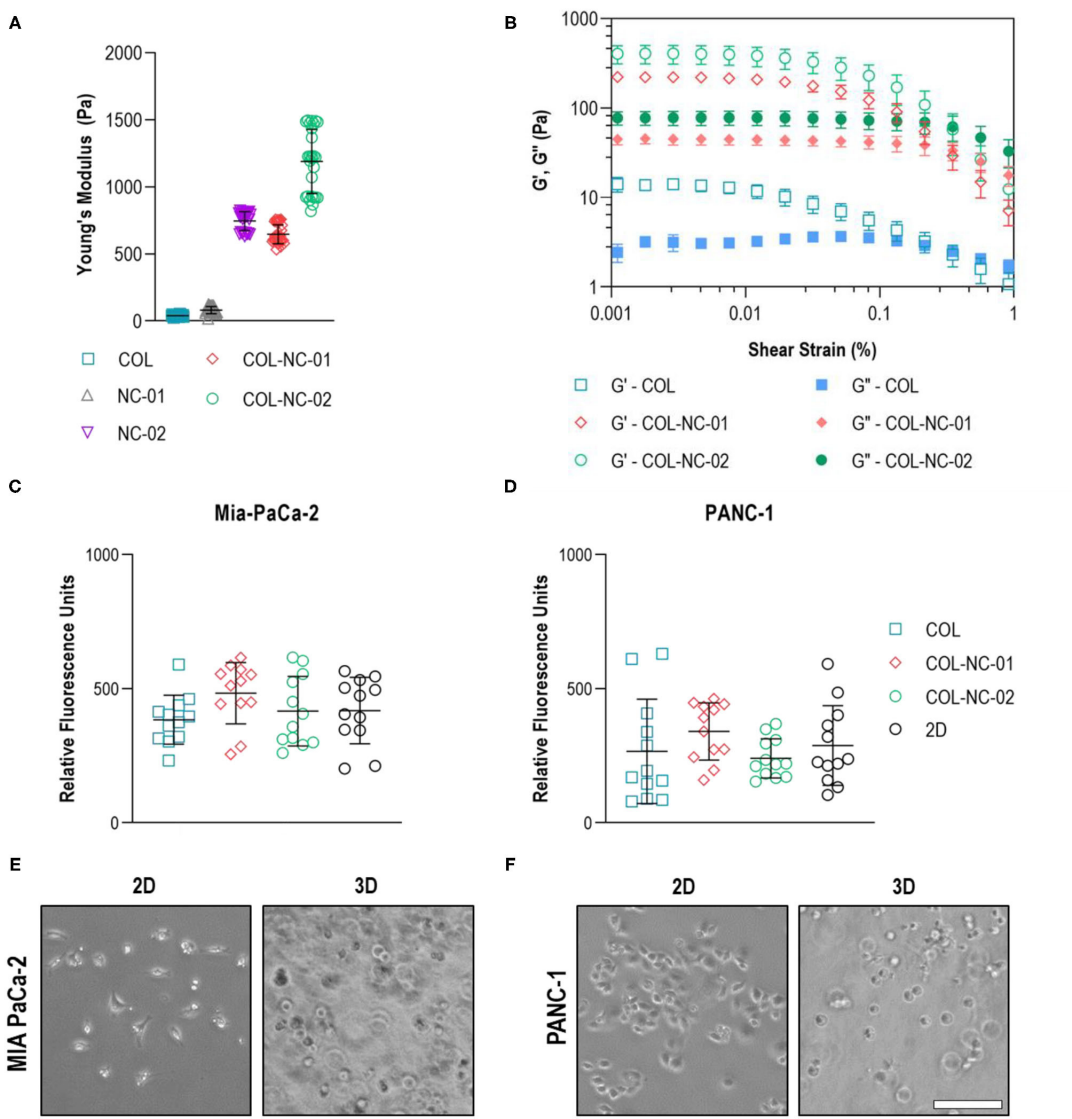
Hydrogel and cell characterisation. **(A)** The reinforcing effect of nanocellulose and collagen on the mechanical properties of the hydrogels represented by the Young's modulus. **(B)** Oscillatory strain sweep measurements indicated that both storage and loss moduli of collagen-nanocellulose hydrogels rose by more than 10-fold, whilst the yield point was unchanged. G', storage modulus; G”, loss modulus. The metabolic activity of MIA PaCa-2 **(C)** and PANC-1 **(D)** cells was unchanged when grown embedded in different hydrogels and as 2D monolayers. MIA PaCa-2 cells **(E)** had a spindle-shaped morphology in 2D and PANC-1 cells **(F)** acquired a polygonal shape, while both cell types were round in 3D. Scale bar = 100 μm. Results shown represent independent experiments performed in triplicates (*n* = 3, error bars = SD). COL, collagen; NC, nanocellulose; COL-NC-01, collagen-nanocellulose at 0.1 wt%; COL-NC-02, collagen-nanocellulose at 0.2 wt%.

The mechanical properties of different hydrogels were measured to determine the matrix which resembled the characteristic stiffness of PDAC tissues. Collagen hydrogels were notably soft, presenting a Young's modulus of 40 ± 7 Pa, whereas nanocellulose hydrogels ranged from 81 ± 26 to 745 ± 68 Pa, depending on the nanofibre concentration. Once collagen was blended with nanocellulose at 0.1 wt%, hydrogels reached a Young's modulus of 647 ± 69 Pa, and those reinforced with 0.2 wt% nanocellulose reached a Young's modulus of 1,189 ± 234 Pa (Figure 2A). Regardless of the presence or absence of nanocellulose, all hydrogels showed a viscoelastic profile, characterised by the storage (G') and loss (G”) moduli curves that indicate their gel-like behaviour, and a similar yield point under 0.2% strain (Figure 2B). Imaging of the matrix structure revealed that collagen hydrogels have larger pores than those of the collagen-nanocellulose hydrogels (Supplementary Figure 1).

To warrant equal cell seeding densities from the start of the 3D cell cultures, the metabolic activity and morphology of the same number of PDAC cells seeded in the 3D matrices and in 2D as reference were assessed after 24 h. Both MIA PaCa-2 and PANC-1 cells showed comparable levels of metabolic activity, indicative of cell viability, when grown embedded in all hydrogel formulations tested. There was no difference in the 3D cell viability, or cell loss, when compared to the 2D reference (Figures 2C,D). Morphological analysis using brightfield imaging showed that MIA PaCa-2 cells had a spindle-shaped morphology and PANC-1 cells acquired a polygonal-like shape when grown in 2D, while cells exhibited a round morphology of similar dimensions within the 3D matrices (Figures 2E,F).

### Proliferation and Morphology of Tumour Spheroids

**Figure 3:**
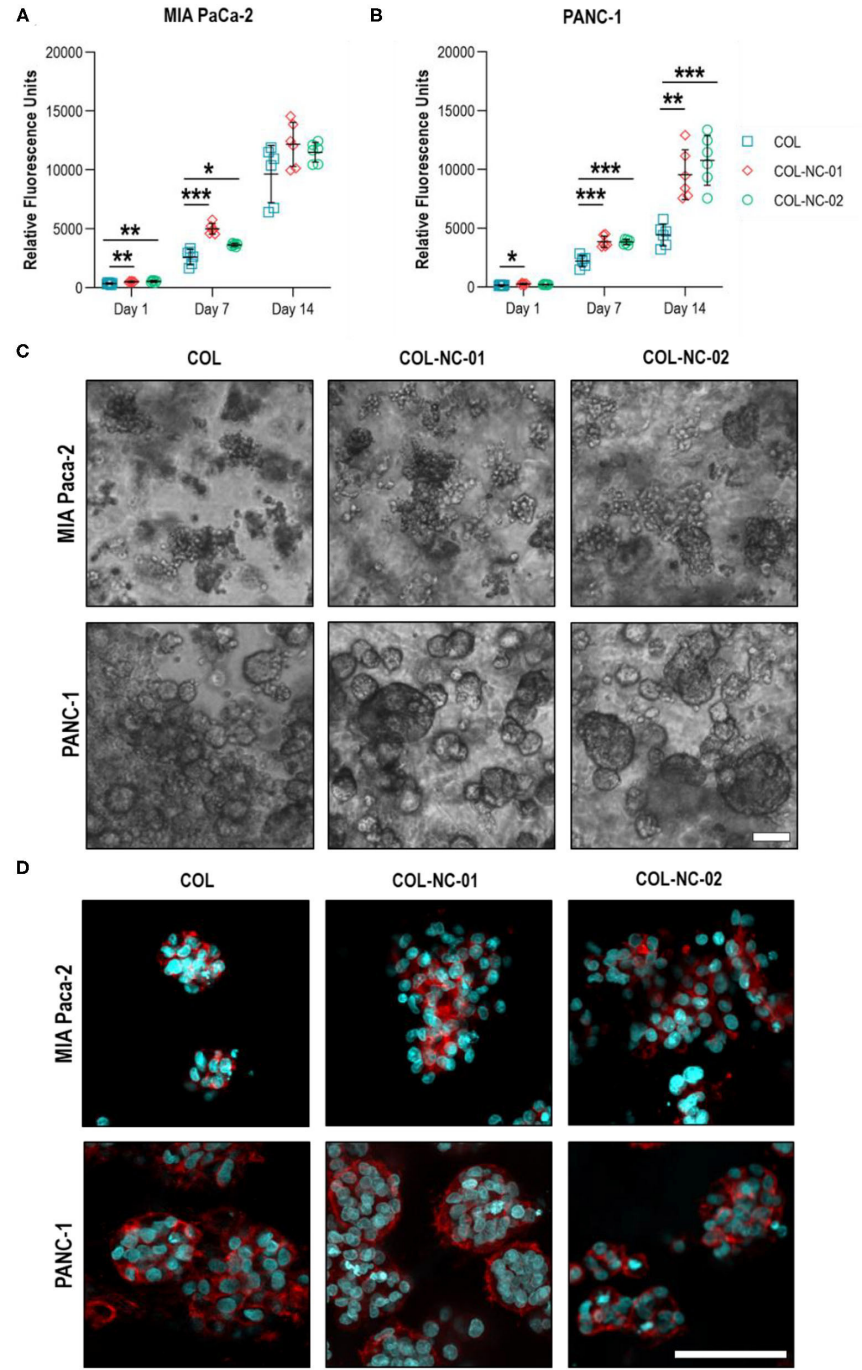
Proliferation and morphology of spheroids formed by PDAC cells. The metabolic activity of MIA PaCa-2 **(A)** and PANC-1 **(B)** cells grown in collagen and collagen-nanocellulose hydrogels progressively increased over the 14 days culture period. **(C)** MIA PaCa-2 cells formed irregular clusters within all hydrogels tested, while PANC-1 cells formed round spheroids with larger diameters with increasing nanocellulose concentration. Scale bar = 100 μm. **(D)** Confocal micrographs of F**-**actin filaments (red) and nuclei (blue) of MIA PaCa-2 and PANC-1 cells in 3D culture. Scale bar = 50 μm. Results shown represent independent experiments performed in triplicates (*n* = 3, error bars = SD; * = *p* ≤ 0.05; ** = *p* ≤ 0.01; *** = *p* ≤ 0.001). COL, collagen; NC, nanocellulose; COL-NC-01, collagen-nanocellulose at 0.1 wt%; COL-NC-02, collagen-nanocellulose at 0.2 wt%.

This is the first study, to our knowledge, using collagen-nanocellulose hydrogels as a 3D model for PDAC, and hence, we sought to assess the proliferation and morphology of MIA PaCa-2 and PANC-1 cells in 3D culture over 14 days. The metabolic activity, indicative of cell proliferation, of tumour spheroids formed in collagen-nanocellulose hydrogels was significantly higher compared to collagen, suggesting enhanced cell proliferation rates. MIA PaCa-2 cells cultured in collagen and collagen-nanocellulose hydrogels had a progressive increase in their metabolic activity, without differences between the nanocellulose-containing matrices (Figure 3A). PANC-1 cells showed a two-fold increased metabolic activity when grown in collagen-nanocellulose hydrogels on days 7 and 14 (Figure 3B). Different from PANC-1, MIA PaCa-2 cells formed spheroids characterised by unshaped clusters, regardless of the matrix composition (Figure 3C). A few uniform spherical morphologies were observed for MIA PaCa-2 cells grown in collagen-nanocellulose hydrogels with increasing mechanical properties. PANC-1 cells formed smaller spheroids in collagen compared to nanocellulose-containing matrices (Supplementary Figure 2), being tightly aggregated in collagen and evenly distributed in nanocellulose-containing matrices (Figure 3C). Immunofluorescent staining of MIA PaCa2 and PANC-1 cells and confocal microscopy confirmed the presence of F-actin filaments and the observed morphologies (Figure 3D).

### Tumour Spheroid Responses to Anti-cancer Treatment

**Figure 4:**
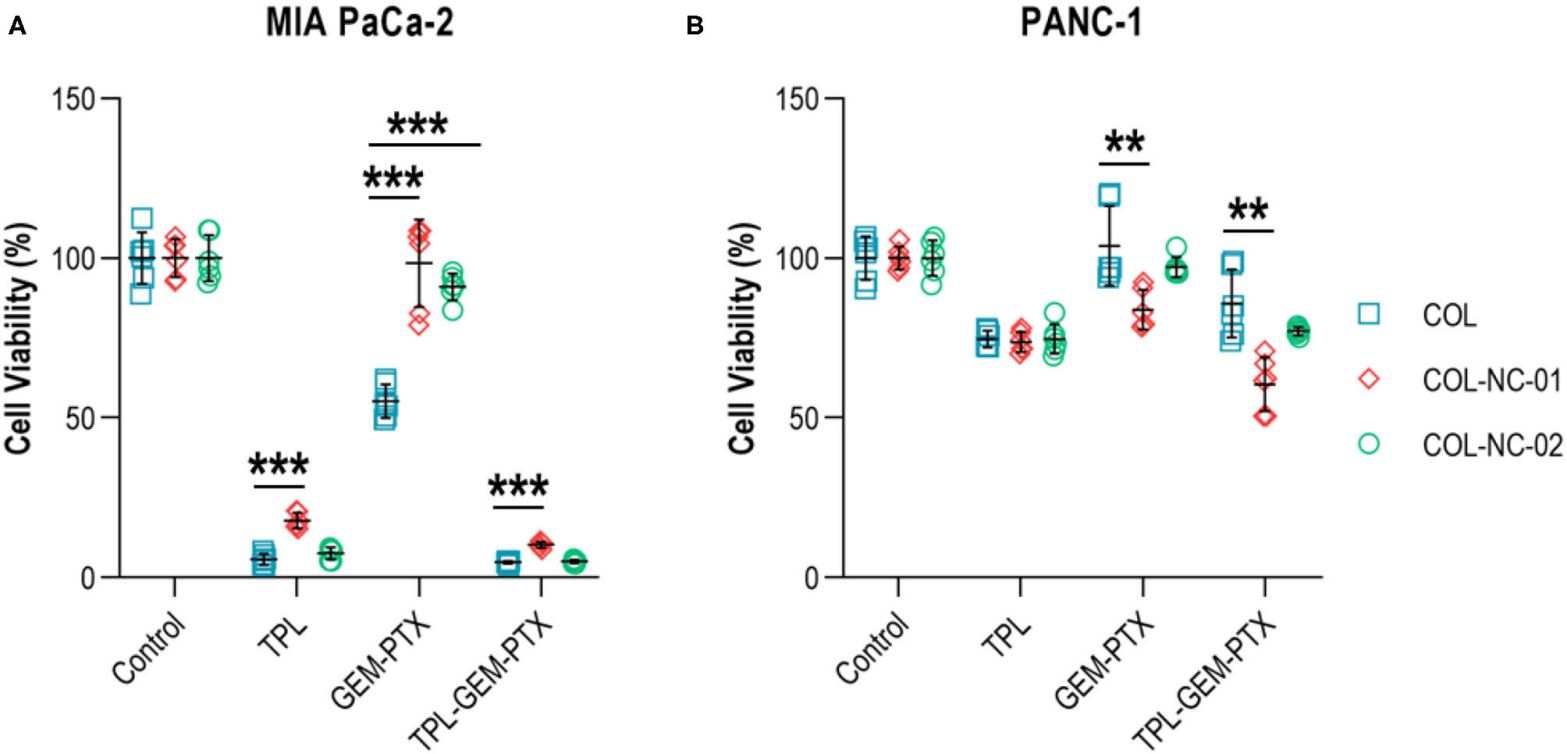
Treatment of tumour spheroids. **(A)** The viability of MIA PaCa-2 cells was reduced about 85% upon treatment with triptolide in the collagen matrix, whereas the treatment with gemcitabine and paclitaxel reduced cell viability by nearly 50%. **(B)** The viability of PANC-1 cells grown in collagen and collagen-nanocellulose matrices was decreased by 30% when exposed to triptolide, while the treatment with gemcitabine and paclitaxel did not have significant cytotoxic effects. Cells were treated with triptolide for 3 days, followed by gemcitabine and paclitaxel for 4 days. Results shown represent independent experiments performed in triplicates (*n* = 3, error bars = SD; ** = *p* ≤ 0.01; *** = *p* ≤ 0.001). COL, collagen; NC, nanocellulose; COL-NC-01, collagen-nanocellulose at 0.1 wt%; COL-NC-02, collagen-nanocellulose at 0.2 wt%; TPL, triptolide; GEM-PTX, gemcitabine-paclitaxel, TPL-GEM-PTX, triptolide-gemcitabine-paclitaxel.

To validate our new 3D cancer model, we determined the responses of tumour spheroids to the treatment combination of gemcitabine and paclitaxel, as well as the addition or absence of the anti-cancer compound triptolide. Triptolide was highly effective in reducing the cell viability of MIA PaCa-2 cells by more than 85% compared with untreated controls. However, the exposure of MIA PaCa-2 cells to gemcitabine and paclitaxel resulted in a cell viability of 55% in the collagen matrix, whereas the cell viability was 98% in collagen-nanocellulose hydrogels. The combined treatment of gemcitabine, paclitaxel, and triptolide reduced the viability of MIA PaCa-2 cells by up to 90% (Figure 4A). The viability of PANC-1 cells grown in any of the 3D matrices exposed to triptolide had a decrease of 30%, while gemcitabine and paclitaxel did not have any effect. The combined treatment of gemcitabine, paclitaxel, and triptolide decreased the viability of PANC-1 cells by 20% in the collagen matrix, whereas the cell viability was reduced by 45 and 30%, respectively in the collagen-nanocellulose hydrogels (Figure 4B).

### Multicellular 3D Cultures of PDAC Cells With Stromal Cell Populations

**Figure 5:**
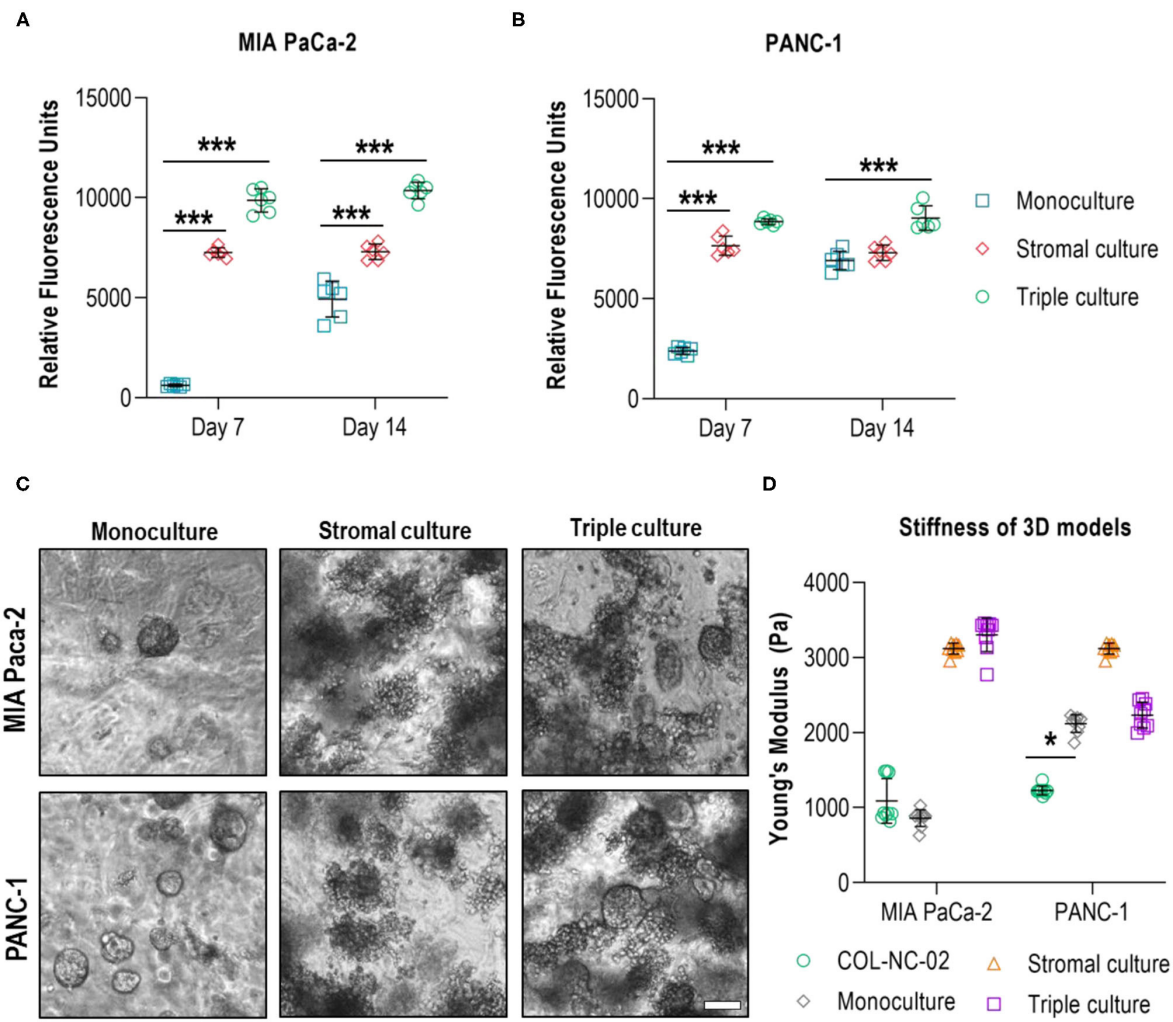
Evaluation of multicellular 3D cultures of tumour spheroids with stromal cells. The metabolic activity of MIA PaCa-2 **(A)** and PANC-1 **(B)** cell monocultures was initially lower than in the stromal and triple cultures in the collagen-nanocellulose matrices and increased over time. **(C)** Fewer spheroids were formed by MIA PaCa-2 cells in both 3D culture settings, while more spheroids were formed by PANC-1 cells. **(D)** The Young's moduli of the MIA PaCa-2 and PANC-1 triple cultures reached 3,303 ± 226 Pa and 2,233 ± 172 Pa, respectively. Results shown represent independent experiments performed in triplicates (*n* = 3, error bars = SD). Scale bar = 100 μm.

To incorporate cellular elements of the pancreatic TME, we co-cultured PDAC cells together with the characteristic stromal cell types, CAFs and myeloid cells, in 0.2 wt% collagen-nanocellulose matrices over 14 days. Total cell numbers across groups were identical. Once the tumour spheroids were formed, the metabolic activity of MIA PaCa-2 and PANC-1 triple cultures with stromal cells was 10 and 4 times higher, respectively, compared to their respective monocultures. On day 14, the metabolic activity of the triple cultures was comparable to day 7, while the metabolic activity increased by up to 3 times in the PANC-1 and Mia PaCa-2 cell monocultures. The stromal cultures had a slightly lower metabolic activity compared to the triple cultures, which did not change during the culture period, demonstrating the growth-promoting effect of the stromal-cancer cell interactions (Figures 5A,B). MIA PaCa-2 cells formed a few round spheroids in monoculture and triple culture, whereas PANC-1 cells formed more spheroids in both 3D culture settings. Several unshaped clusters were formed by the stromal cells (Figure 5C). The Young's modulus of the MIA PaCa-2 cell monocultures slightly decreased to 859 ± 112 Pa compared to the cell-free collagen-nanocellulose matrix. Once the stromal cells were grown together with MIA PaCa-2 cells, the Young's modulus reached 3,303 ± 226 Pa. Matrices seeded with PANC-1 cells presented a Young's modulus of 2,119 ± 118 Pa, and those seeded with PANC-1 and stromal cells reached 2,233 ± 172 Pa. Matrices containing stromal cultures had a 3 times increased Young's modulus of about 3,118 ± 74 Pa compared to MIA PaCa-2 and PANC-1 cell monocultures (Figure 5D).

### The Effect of Anti-cancer Treatment on Multicellular 3D Cultures and Matrix Stiffening

**Figure 6:**
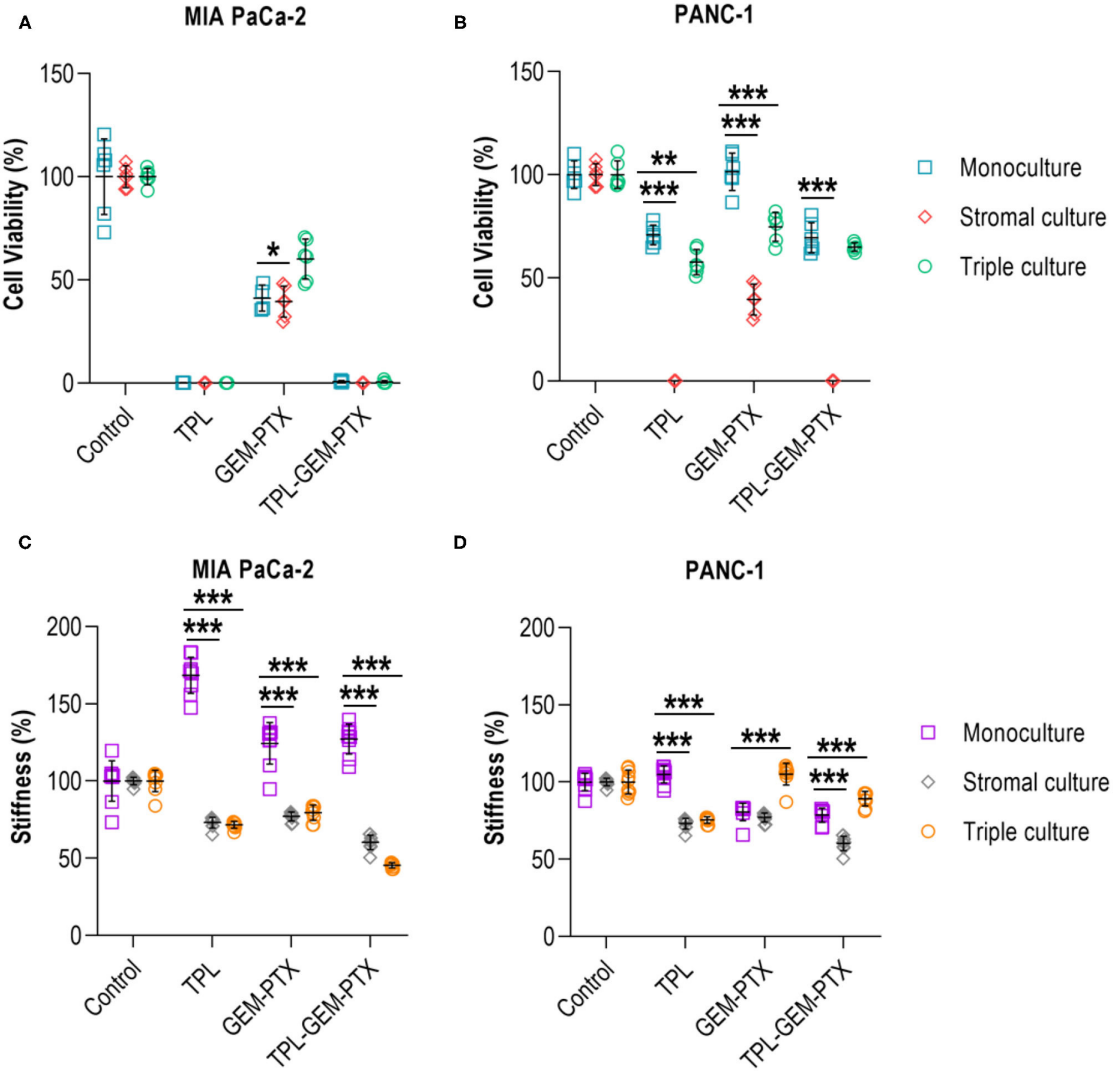
Treatment of multicellular 3D cultures. **(A)** Triptolide treatment was effective in reducing the cell viability of MIA PaCa-2 monoculture, stromal and triple cultures. No additive effect was achieved by combining triptolide with gemcitabine and paclitaxel. **(B)** PANC-1 monoculture and triple culture had a decreased cell viability by 30-40% when exposed to triptolide and in combination with gemcitabine and paclitaxel. **(C)** Treatment with triptolide, gemcitabine, and paclitaxel increased the mechanical properties of MIA PaCa-2 cell monocultures but reduced the mechanical properties of stromal and triple cultures. **(D)** PANC-1 cell monocultures and stromal cultures had reduced mechanical properties upon gemcitabine and paclitaxel treatment, whereas triple cultures showed a reduction of the mechanical properties upon triptolide exposure. Cells were treated with triptolide for 3 days, followed by gemcitabine and paclitaxel for 4 days. Results shown represent independent experiments performed in triplicates (*n* = 3, error bars = SD; ** = *p* ≤ 0.01; *** = *p* ≤ 0.001). TPL, triptolide; GEM-PTX, gemcitabine-paclitaxel; TPL-GEM-PTX, triptolide-gemcitabine-paclitaxel.

Finally, we wanted to investigate whether the incorporation of the stromal cell populations of the pancreatic TME had any effect on the treatment responses and mechanical properties of the 0.2 wt% collagen-nanocellulose matrices. The multicellular 3D cultures were exposed to triptolide followed by the combined treatment with gemcitabine and paclitaxel, and the metabolic activities and Young's moduli were determined. Treatment with triptolide had a maximal effect on cell viability in all 3D cultures using MIA PaCa-2 cells, while PANC-1 cell monocultures had a reduced metabolic activity of 30% (Figures 6A,B). The treatment with gemcitabine and paclitaxel did not affect the PANC-1 cell monoculture, but triggered a response of 60% in the MIA PaCa-2 cell monoculture. The viability of the triple cultures was reduced by 45% upon combined gemcitabine and paclitaxel treatment. There was no enhanced effect when combining triptolide with both chemotherapeutics. The Young's modulus of the matrix containing MIA PaCa-2 cell monocultures increased up to 10% upon treatment with triptolide (Figure 6C). Treatment with gemcitabine and paclitaxel softened the matrices with the stromal and triple cultures with MIAPaCa-2 cells by 15%. Similarly, the exposure to triptolide combined with gemcitabine and paclitaxel reduced the Young's moduli of the stromal and triple cultures by 42%. The treatment with triptolide had no effect on the Young's modulus of the PANC-1 cell monoculture and reduced the Young's moduli of the stromal and triple cultures by 30% (Figure 6D). The treatment with gemcitabine and paclitaxel decreased the Young's moduli of the PANC-1 cell monoculture and stromal culture also by 30%, with only minor effects in the triple culture.

## Discussion

The pancreatic TME is characterised by a highly fibrotic tissue constituted by a collagen-rich ECM and various stromal cells. Mostly CAFs are responsible for secreting large amounts of collagen, which is remodelled and crosslinked to other ECM proteins, resulting in the stiffening of the matrix (36, 40). These changes in the mechanical properties, or stiffness, of the TME trigger an altered mechano-transduction and signalling pathways associated with cell viability, proliferation, and invasion, which prompts the aggressive behaviour of pancreatic cancer cells (4, 20). The fibrotic tissue prevents chemotherapeutics from reaching their target cells, leading to therapy resistance and cancer progression, which ultimately has a negative impact on survivorship (3). There is a concerted effort to understand the contribution of the extracellular and cellular elements of the pancreatic TME and to find alternative therapeutic strategies (15, 41). In this context, bioengineered TME models have emerged as modern 3D approaches to study the tumour biology of this disease, reproducing some elements of the complex interaction between the different cell populations and the stiff matrix.

Bioengineered 3D cancer models are superior to traditional 2D monolayer cultures because of the use of biomaterials to recreate the mechanical and chemical properties of the tumour-specific ECM. Having a 3D architecture that provides the desired mechanical stimuli within a desired range, biomaterials are designed to represent a controllable microenvironment with physiological parameters, including pH, osmotic pressure and ionic strength (42, 43). Collagen matrices are commonly used to replicate some of the physicochemical and biological conditions required for 3D cultures, such as integrin binding sites that are critical for mechano-transduction (44). However, when assembled in a 3D structure, collagen forms a matrix with poor mechanical properties, limited to a soft or low range. In contrast, nanocellulose is an inert biocompatible polymer that forms a tailorable matrix that not only varies in its mechanical properties, but also chemically *via* the incorporation of different functional groups (45). Due to the high affinity between collagen and nanocellulose (42), we have previously blended collagen with nanocellulose hydrogels to grow tissue-derived organoids (35). In this study, we explored for the first time, to our knowledge, collagen-nanocellulose hydrogels as a 3D matrix to model the pancreatic TME.

We found that the mechanical properties of the hydrogel were modulated by the concentration of nanocellulose fibres. From a biomaterial perspective, the interaction between collagen and nanocellulose relies on the formation of hydrogen bonds and van der Waals bonds between both polymers; this mechanism is similar to collagen and other ECM proteins. Indeed, the adhesion energy between nanocellulose and collagen was reported to be similar to that between collagen and laminin or between different types of collagen (46). For this reason, the blended, or composite, matrices reached a stiffness range that was not achievable with collagen alone. Despite the maximum Young's modulus of 1,189 ± 234 Pa for the collagen-nanocellulose matrix, this is still below the levels reported in literature for PDAC tissue (19–21). Upon incorporation of PDAC and stromal cells into the collagen-nanocellulose matrix, the Young's modulus increased to 3,303 ± 226 Pa, demonstrating the importance of the stromal cells for matrix stiffening. This effect was corroborated by matrices seeded with stromal cells only, which increased the Young's modulus to about 3,118 ± 74 Pa. Stromal cells are considered the main contributor to the secretion of ECM elements as indicated by the increased deposition of collagens derived from CAFs. This leads to the formation of fibrotic tissue with a modified 3D architecture and aligned collagen fibres and enhances the stiffness observed in pancreatic tumours (47–49). Therefore, our results confirm the suitability of 3D collagen-nanocellulose matrices to recreate the mechanical properties of the TME within the lower range of PDAC tissues.

The formation of tumour spheroids in collagen-nanocellulose hydrogels was assessed using the two representative PDAC cell lines, MIA PaCa-2, and PANC-1. Our findings are linked to the cell phenotype. While MIA PaCa-2 have a mesenchymal phenotype, PANC-1 cells present an intermediate profile, displaying an epithelial phenotype with some mesenchymal-like aspects (50, 51). Although both PDAC cells formed spheroids and proliferated when grown embedded on our matrices, the motility and evasion of the MIA PaCa-2 cells was evidenced, resulting in irregular spheroids and cells eventually escaping the matrix. Conversely, PANC-1 cells remained adhered to the hydrogels and formed round spheroids. The higher level of metabolic activity measured in PANC-1 spheroids grown in the collagen-nanocellulose matrices is in line with other reports (21).

We validated our new 3D cancer model using the anti-cancer compound triptolide and the chemotherapeutics gemcitabine and paclitaxel. Triptolide is a plant-based substance with anti-cancer properties against several cancer types, including pancreatic tumours (52), by inducing apoptosis in stromal cells. Indeed, studies have shown that triptolide modulates the expression of regulatory transcription factors by CAFs, preventing cell proliferation and secretion of ECM proteins like collagens and hyaluronic acid (53, 54). Gemcitabine is a cytotoxic drug administered to patients diagnosed with PDAC, in combination with nanoparticle albumin-bound paclitaxel (nab-paclitaxel), resulting in improved survival rates compared to monotherapy (55). However, due to the emergence of multi-drug resistance in patients with PDAC (56), gemcitabine and nab-paclitaxel have been combined with other compounds, in particular those which target the fibrotic and dense ECM to improve the delivery of cytotoxic drugs (57). In a step-wise strategy, we first assessed the effects of triptolide and gemcitabine combined with paclitaxel in PDAC cell monocultures grown in different hydrogels and found that triptolide was effective against MIA PaCa-2 cells but not PANC-1 cells. These differential responses may be explained by the anti-proliferative effects of triptolide on mesenchymal-like cells and an epithelial-mesenchymal transition (58). In contrast, gemcitabine and paclitaxel treatment exhibited lower efficacy in the PDAC cell monocultures, in particular MIA PaCa-2 cells, in the stiffer collagen-nanocellulose matrices compared to the soft collagen matrix. Our results corroborate reports that showed the lowered anti-cancer activity of these cytotoxic drugs in PDAC models using stiff matrices (59, 60). Thus, collagen-nanocellulose matrices were used for multicellular 3D cultures that included the two stromal cell populations, CAFs and myeloid cells.

In the triple cultures, triptolide targeted the stromal cells by reducing their cell viability and the mechanical properties. As with the PDAC cell monocultures, triptolide had different anti-cancer activities in MIA PaCa-2 and PANC-1 cells, which translated into cell death and <30% cell viability, respectively. Our findings are supported by other reports that demonstrate that triptolide acts as a cytotoxic agent for both pancreatic cancer cells and CAFs, by disrupting transcription factors that mediate the expression of regulatory genes (53). Besides triptolide, other compounds like blebbistatin and fasudil have also been used in 3D models to target CAFs and stromal remodelling in the pancreatic TME (61).

In contrast, treatment with gemcitabine and paclitaxel showed limited cytotoxicity in our 3D cancer model. Triple cultures had a 50% cell viability, and the mechanical properties of the collagen-nanocellulose matrix was not significantly changed. Chemoresistance of pancreatic tumours to gemcitabine has been associated with the tumour immune microenvironment (62), herein represented by the myeloid cells. The treatment with triptolide combined with gemcitabine and paclitaxel of the triple cultures exemplified the improved outcomes of therapeutic combination approaches. Indeed, once triptolide targeted the stromal cells and reduced the matrix stiffness by more than 55%, the chemotherapeutics were able to act on the MIA PaCa-2 cells. However, in the PANC-1 triple cultures, the pre-treatment with triptolide did not result in an enhanced effect of gemcitabine and paclitaxel, and the matrix retained 92% of its stiffness. Apart from the differential effects of triptolide on mesenchymal-like and epithelial cells, we note here a possible chemical interaction between triptolide, gemcitabine, and paclitaxel with the nanocellulose fibres in our matrix. This hypothesis is supported by the use of nanocellulose-based biomaterials as carriers for drug delivery (63). Similarly, hydrogels loaded with paclitaxel demonstrated the affinity between nanocellulose and paclitaxel (27, 64), which may prevent drug diffusion into the tumour spheroids within the matrix and may interfere with the mechanical properties. This could explain the increased mechanical properties of the MIA PaCa-2 cell monocultures and the contrary results for the PANC-1 cells.

## Conclusion

Collagen-nanocellulose hydrogels are a suitable 3D matrix to resemble some of the elements of the pancreatic TME. The mechanical properties of the 3D matrix are tuneable to achieve a characteristic range of stiffness of PDAC tissues. Tumour spheroids formed within 7 days and allow the testing of different treatment and combination strategies. The compatibility between collagen and nanocellulose can be further explored by integrating other ECM elements, such as hyaluronan or laminin. The multicellular 3D cancer model may be used to study tumour initiation and progression to metastasis, as well as to screen novel or improved treatments for PDAC.

## Data Availability Statement

The raw data supporting the conclusions of this article will be made available by the authors, without undue reservation.

## Author Contributions

RC and DL designed the study. RC performed all the experiments and wrote the manuscript. MA and AF established the cancer-associated fibroblasts. VK, GG, and DL revised the manuscript. All authors contributed to the article and approved the submitted version.

## Conflict of Interest

The authors declare that the research was conducted in the absence of any commercial or financial relationships that could be construed as a potential conflict of interest.

## Publisher's Note

All claims expressed in this article are solely those of the authors and do not necessarily represent those of their affiliated organizations, or those of the publisher, the editors and the reviewers. Any product that may be evaluated in this article, or claim that may be made by its manufacturer, is not guaranteed or endorsed by the publisher.
