# Supplementary material for: 3D Collagen-Nanocellulose Matrices Model the Tumour Microenvironment of Pancreatic Cancer
Source: Front Digit Health. 2021 Jul 26;3:704584. doi: 10.3389/fdgth.2021.704584 (PMC8521838; doi:10.3389/fdgth.2021.704584)
Supplement: Supplementary file 1 [file Data_Sheet_1.PDF]

**3D collagen-nanocellulose matrices model the tumour microenvironment of pancreatic cancer**

Rodrigo Curvello[1], Verena Kast[2], Mohammed H. Abuwarwar[3], Anne L. Fletcher[3], Gil Garnier[1,4], Daniela Loessner[1,5,6]

[1]Department of Chemical Engineering, Faculty of Engineering, Monash University, Clayton, Victoria 3800, Australia

[2]Leibniz Institute of Polymer Research Dresden e.V., Max Bergmann Center of Biomaterials Dresden, Hohe Straβe 6, 01069, Dresden, Germany

[3]Department of Biochemistry and Molecular Biology, Biomedicine Discovery Institute, Monash University, Clayton, Victoria, 3800, Australia

[4]Bioresource Processing Research Institute of Australia (BioPRIA), Department of Chemical Engineering, Monash University, Clayton, Victoria, 3800, Australia

[5]Department of Materials Science and Engineering, Faculty of Engineering, Monash University, Clayton, Victoria 3800, Australia

[6]Department of Anatomy and Developmental Biology, Biomedicine Discovery Institute, Faculty of Medicine, Nursing and Health Science, Monash University, Clayton, Victoria 3800, Australia

*Corresponding author: daniela.loessner@monash.edu

**Supplementary Information**

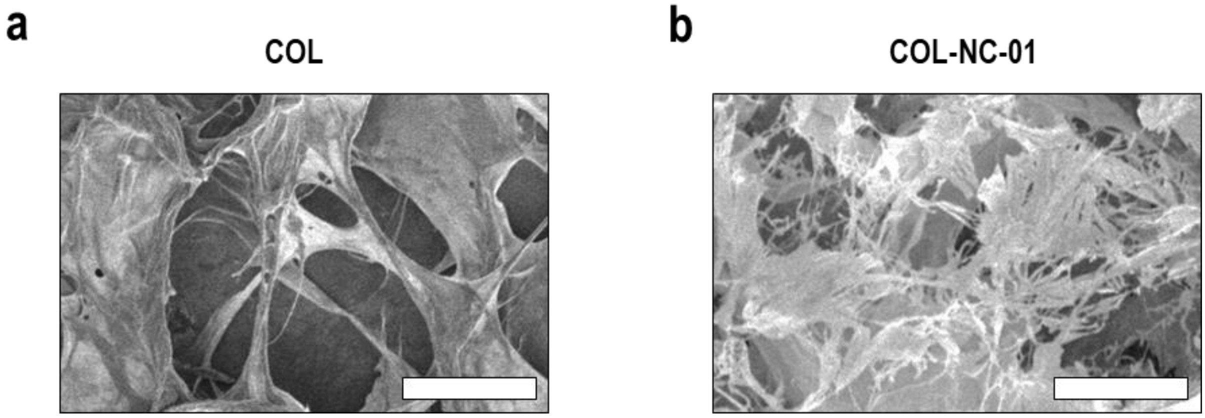


**Figure S1. Structure of collagen and collagen-nanocellulose hydrogels. (a)** COL and **(b)** COL-NC-01 hydrogels had a three-dimensional fibrous structure. Scale bars = 100 µm. COL, collagen; COL-NC -01, collagen-nanocellulose at 0.1 wt%.

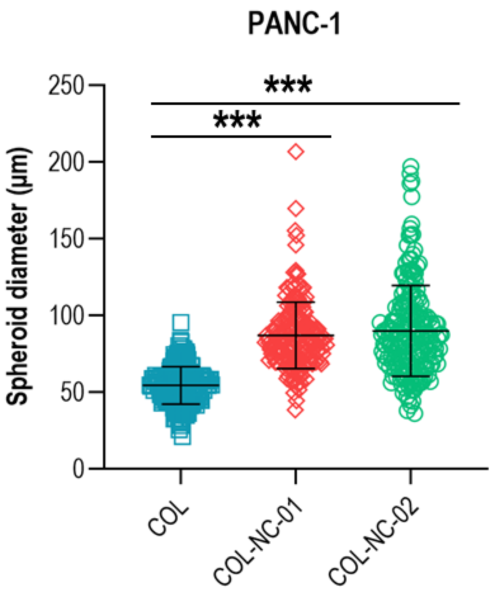


**Figure S2. Quantification of the diameter of PANC-1 spheroids.** PANC-1 spheroids grown in collagen hydrogels had a diameter of 54 ± 12 µm, whereas those embedded in collagen-nanocellulose matrices had a diameter of 87 ± 22 µm (COL-NC-01) and 90 ± 30 µm (COL-NC-02), respectively. Results shown represent multiple measurements obtained of independent experiments performed in triplicates (n = 200, error bars = SD, *** = p ≤ 0.001). COL, collagen; COL-NC-01, collagen-nanocellulose at 0.1 wt%; COL-NC-02, collagen-nanocellulose at 0.2 wt%.
